# Development of an Evidence-Informed Blog to Promote Healthy Eating Among Mothers: Use of the Intervention Mapping Protocol

**DOI:** 10.2196/resprot.7147

**Published:** 2017-05-19

**Authors:** Audrée-Anne Dumas, Simone Lemieux, Annie Lapointe, Véronique Provencher, Julie Robitaille, Sophie Desroches

**Affiliations:** ^1^ Institute of Nutrition and Functional Foods Laval University Quebec City, QC Canada

**Keywords:** blogs, healthy eating, knowledge translation, theory-driven design, intervention mapping, clinical research protocol

## Abstract

**Background:**

Low adherence to dietary guidelines and a concurrent rise of obesity-related chronic diseases emphasize the need for effective interventions to promote healthy eating. There is growing recognition that behavior change interventions should draw on theories of behavior change. Online interventions grounded in theory lead to increased effectiveness for health behavior change; however, few theory-driven social media-based health promotion interventions have been described in the literature.

**Objective:**

The objective of this study was to describe the application of the Intervention Mapping (IM) protocol to develop an evidence-informed blog to promote healthy eating among French-Canadian mothers of preschool and school-aged children.

**Methods:**

The following six steps of the IM protocol were performed. In Step 1, a preliminary needs assessment included a literature search on theoretical domains predicting Vegetables and Fruits intakes and Milk and Alternatives intakes in adults (ie, knowledge, beliefs about capabilities, beliefs about consequences, intention/goals) and a qualitative study including focus groups to identify female Internet users’ perceptions of their use of healthy eating blogs. In Step 2, two behavioral outcomes were selected (ie, increase daily intakes of Vegetables and Fruits and Milk and Alternatives of mothers to reach Canadian dietary recommendations) and subsequently divided into six performance objectives inspired by national and international dietary recommendations such as planning for healthy meals. A matrix of change objectives was then created by crossing performance objectives with theoretical domains predicting Vegetables and Fruits intakes and Milk and Alternatives intakes in adults. Step 3 consisted of selecting theory-based intervention methods (eg, modeling and goal setting) and translating them into practical applications for the context of a dietary intervention delivered through a blog. A 6-month intervention was developed in Step 4 in which we aimed to address one performance objective per month in weekly blog publications written by a registered dietitian. For Step 5, we sought to include engagement-promoting methods (eg, peer and counselor support) to promote mothers’ use of the blog and adherence to the intervention. Finally in Step 6, a randomized controlled trial has been launched to evaluate the effects of the blog on dietary behaviors of French-Canadian mothers.

**Results:**

The intervention study is expected to be completed in March 2018.

**Conclusions:**

An intervention mapping protocol allowed for effective decision making in the development of a novel knowledge translation tool to increase adherence to dietary recommendations among mothers of preschool and school-aged children.

## Introduction

### Low Adherence to Dietary Recommendations

Lifestyle modifications—including the consumption of a balanced diet—can reduce obesity and the risk of obesity-related chronic diseases despite minimal or no weight loss [1]. Many health agencies thus recommend adopting a healthy diet to prevent obesity-related chronic diseases [2-4]. As part of a healthy diet, Vegetables and Fruits as well as Milk and Alternatives food groups are components of the Canadian adaptation of the Healthy Eating Index [5], which measures conformity to Canada’s Food Guide. Examples of Milk Alternatives included in Canada’s Food Guide are buttermilk, cheese, fortified soy beverages, kefir, paneer, pudding/custard made with milk, yogurt, and yogurt drinks [6]. Higher intakes of vegetables and fruit [5], as well as dairy product consumption [7] have been associated with improved diet quality and reduction in the risk of cardiovascular-related clinical outcomes in adults in epidemiologic studies. Despite various healthy eating public health initiatives, fewer than 50% of Canadian adults and children reach daily recommended intakes for the Vegetables and Fruits and Milk and Alternatives food groups [8]. This gap between nutrition recommendations and population dietary habits emphasizes the need to develop effective knowledge translation strategies to help individuals improve their diets.

### Social Media Platforms as Knowledge Translation Tools to Promote Health Behavior Change

Social media represent an exciting strategy for health care professionals to improve knowledge translation to Internet users. Social media are Web-based platforms devoted to blogging, social networking, collaborative writing projects, and wikis [9]. Studies have shown potential effectiveness of social media interventions on health outcomes, such as a decrease in dietary fat consumption in adults [10,11]. According to a systematic review conducted by Webb et al [12], the effectiveness of Web-based interventions promoting health behavior change is enhanced by a more extensive use of theory and the incorporation of behavior change techniques. However, this review did not study interventions delivered through social media sites and currently few theory-informed social media‒based interventions in health promotion have been described in the literature [13,14].

Among the different types of social media, emerging evidence revealed that blogs are used by mothers notably for seeking information about child-feeding practices [15]. Blogs consist of Web-based personal journals with dated entries (posts) displayed in reverse chronological order [9]. The interactive communication between users and experts, such as registered dietitians (RDs), through blogs could improve knowledge translation in nutrition to support dietary behavior change efforts [16]. While blogs could foster dietary behavior change among mothers through enhanced knowledge translation, they have never been studied in that context.

### Aim of this Study

The objective of this study was to describe the use of the Intervention Mapping (IM) protocol [17] to develop an evidence-informed blog written by an RD—used as a knowledge translation tool—to promote healthy eating among French-Canadian mothers of preschool and school-aged children.

## Methods

### Overview

The IM protocol [17] is a planning framework for developing evidence-based health programs through an ecological approach. IM protocol guides the selection of relevant theories to improve the likelihood of effectiveness in health behavior interventions [18].

**Figure 1:**
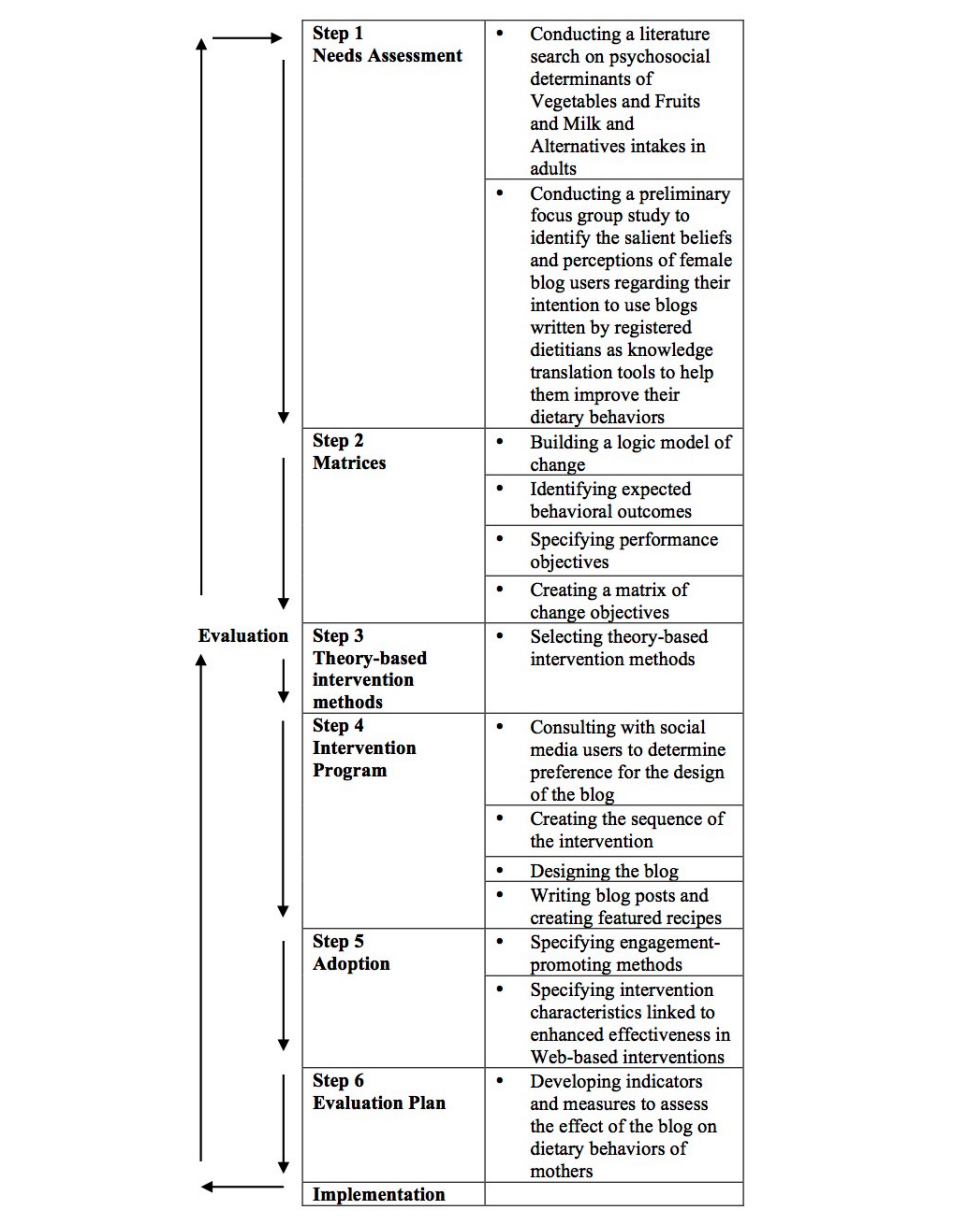
Intervention mapping steps and tasks (adapted from Bartholomew et al).

This study followed six distinct steps inspired by IM protocol: (1) conducting a needs assessment through a literature search, and a preliminary focus group study, (2) specifying expected behavior outcomes and performance objectives for the intervention, (3) selecting theory-based intervention methods, (4) producing program components and materials, (5) planning for the adoption, and (6) the evaluation of the intervention blog (Figure 1).

### Step 1: Conducting a Needs Assessment

The aim of the first step of IM protocol was to assess the health problem and its impact on quality of life, the behavioral and environmental causes of the problem, and the determinants associated with these health-risk causes [17]. For this step, we performed a literature search of psychosocial determinants of Vegetables and Fruits and Milk and Alternatives intakes in adults, in January 2015, and conducted a preliminary focus group study [19], as described below.

#### Literature Search

According to a systematic review performed by Guillaumie et al [20], the most consistent psychosocial determinants predicting the consumption of Vegetables and Fruits in adults in cross-sectional and longitudinal studies were habit, intentions or goals, beliefs about consequences, and knowledge, with the addition of taste for vegetable consumption. In cross-sectional studies [21-24], the most consistent psychosocial determinants predicting the consumption of Milk and Alternatives were beliefs about consequences, beliefs about capabilities, and intentions or goals. We classified these psychosocial determinants according to the validated Theoretical Domains Framework [25]—an integrative framework of 12 theoretical domains derived from theories of behavior change [26].

#### Preliminary Focus Groups Study

Prior to measuring the impact of a healthy eating blog on health outcomes, user views and perceptions toward the use of such blogs to improve dietary habits should first be identified to guide the development of future interventions. Thus, as a second step of the needs assessment, we conducted a preliminary focus group study to identify the salient beliefs and perceptions of female blog users regarding their intention to use blogs written by RDs as knowledge translation tools to improve their dietary behaviors. Methodological details of this study are provided elsewhere [19]. In brief, 33 French-Canadian women with a mean age of 44 years old completed the study that was conducted between April and June 2013. Data from this study showed that women preferred blogs that clearly identified the RD blogger (ie, name, picture, academic education, and professional expertise), that were visually attractive and well structured, that included recipes for putting dietary recommendations given by the RD blogger in practice, and that were updated with new information every week [19]. Women also preferred an RD blogger who supported their messages with references to published scientific papers or to other reliable sources [19].

### Step 2: Preparing Matrices of Change Objectives

The aim of the second step of IM protocol was to state expected outcomes for health-related and environmental conditions in a logic model of change, which represents pathways for intervention effects [17]. Then, a crucial step for IM was the creation of a matrix of change objectives by crossing performance objectives with theoretical domains of health behaviors and writing change objectives. Performance objectives enable an efficient transition from a behavior condition to a detailed description of its components and ensure the appropriateness of the intervention’s behavioral outcomes. Change objectives specify what needs to be achieved in order to accomplish performance objectives.

#### Building the Logic Model of Change

**Figure 2:**
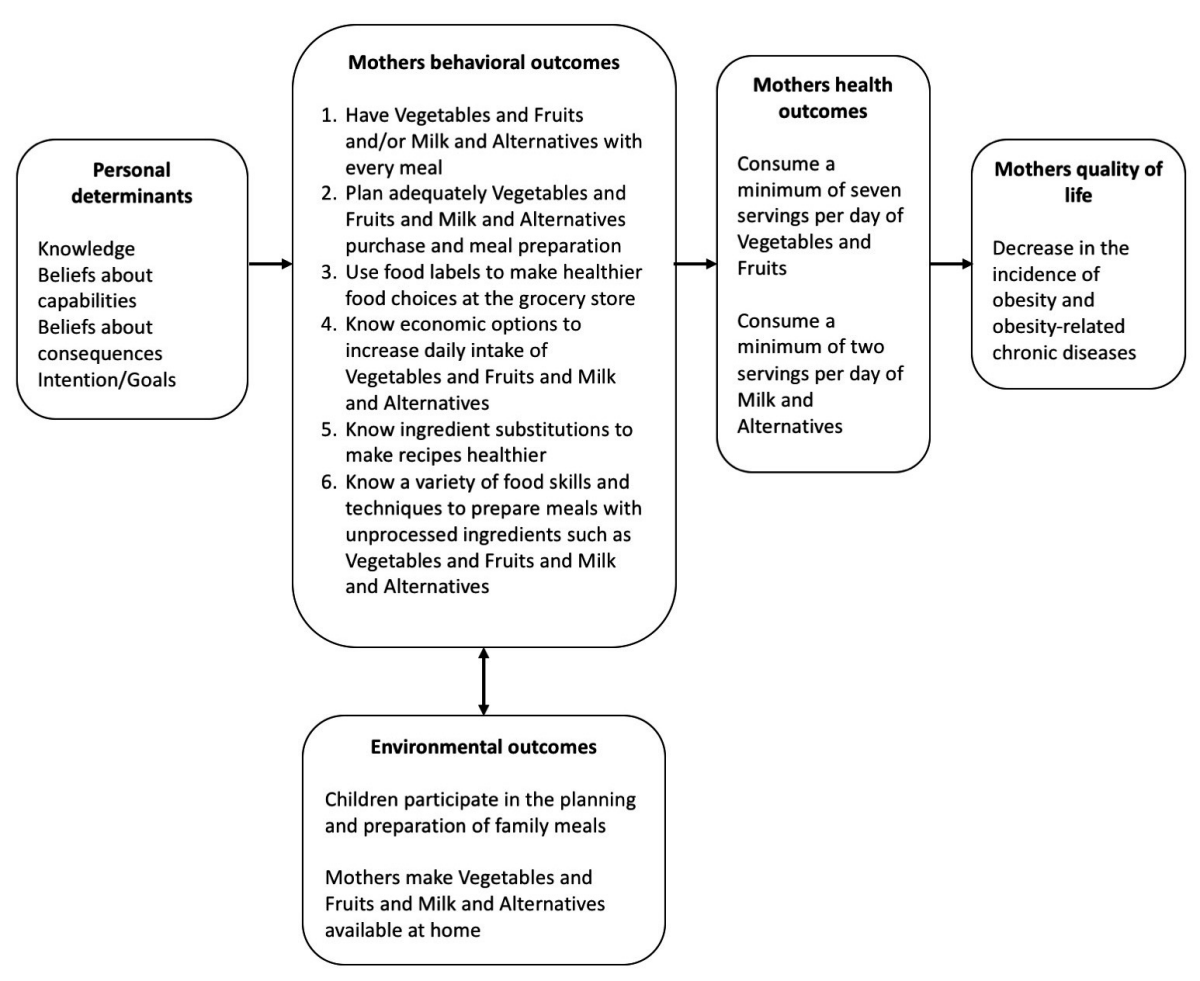
Intervention logic model of change.

First, we developed a logic model of change (Figure 2) based on the results of the needs assessment described in Step 1. Working from right to left, the model starts with the goals for health and quality of life outcomes to be achieved by the intervention. Next, we specified performance objectives for obtaining behavioral outcomes. Performance objectives were then examined in light of the theoretical domains predicting the consumption of Vegetables and Fruits and Milk and Alternatives food groups in adults to generate change objectives. The assumption of this model is that changes in the selected theoretical domains will influence achievement of the performance objectives, which will enable accomplishment of the behavioral outcomes.

#### Identifying Behavioral Outcomes

Behavioral outcomes should state, in terms of behaviors, what should be accomplished as a result of the health promotion program [17]. We formulated two behavioral outcomes for the intervention based on our previous needs assessment: (1) in the next 6 months, mothers will increase their consumption of Vegetables and Fruits to include seven servings per day, and (2) in the next 6 months, mothers will increase their consumption of Milk and Alternatives to include two servings per day.

#### Specifying Performance Objectives

We subdivided the behavioral outcomes into six performance objectives inspired by Health Canada Eat Well Campaign [27], health recommendations of the World Health Organization [28], as well as the perceived barriers of mothers to healthy eating, such as availability and costs of Vegetables and Fruits, and food skills confidence to prepare family meals [29-31]. In chronological order of appearance on the blog, performance objectives were (1) mothers have Vegetables and Fruits and/or Milk and Alternatives with every meal, (2) mothers plan adequately Vegetables and Fruits and Milk and Alternatives purchases and meal preparation, (3) mothers make healthy food choices at the grocery store, (4) mothers know economic food options to increase daily intake of Vegetables and Fruits and Milk Alternatives, (5) mothers increase the daily intake of Vegetables and Fruits and Milk and Alternatives of the family, and (6) mothers make healthy substitutions in recipes (Table 1).

#### Creating a Matrix of Change Objectives

We then created a matrix of change objectives by crossing performance objectives with theoretical domains predicting Vegetables and Fruits intakes and Milk and Alternatives intakes in adults (Table 1).

### Step 3: Selecting Theory-Based Intervention Methods

The aim of the third step of IM protocol was to identify theory-based intervention methods to change the psychosocial determinants of health behaviors, while being sensitive to the target population and consistent with performance objectives [17]. For the selection of these methods, we used the Basic methods for behavior change from the IM taxonomy [33] and the behavior change techniques from the 93-item Behavior Change Technique Taxonomy v1 [34]. Theory-based methods for change are general techniques or processes that have been shown to enable change in one or more determinants of behavior and have their origins in behavioral and social science theories [17]. Behavior change techniques are defined as active components of interventions that aim to change health behaviors [34]. Behavior change techniques were grouped by the theoretical domains of the Theoretical Domains Framework [25], based on the structure proposed by Cane et al [35]. Two authors (A-AD and AL) independently chose theory-based methods that were applicable in the context of an online dietary intervention delivered through a blog [16,36].

Then, theory-based methods were translated into practical applications to the blog (Table 2). Practical applications are defined as specific translation of theory-based methods for practical use in ways that fit the intervention population and the context in which the intervention will be conducted [17]. For example, “modeling” would be an effective technique to use in order to increase mothers’ beliefs about capabilities (self-efficacy). For this task, we took into account theoretical parameters under which theory-based methods are shown to be effective [17]. For example, modeling can be a strong method only when certain parameters are met, for instance, when the participants identify with the model, when the model is reinforced for that particular behavior, and when they expect to be reinforced in a similar way [33]. One application for modeling in the blog was the demonstration by the RD blogger of real-life examples of how she used feasible skills (such as meal planning) to increase her consumption of Vegetables and Fruits and her consumption of Milk and Alternatives every day, and how changing these behaviors improved the quality of her eating habits. The final selection of theory-based methods and practical applications was performed by consensus between 2 authors (A-AD and AL). A third author (SD) was available to resolve conflicts when necessary.

Additionally, we selected techniques inspired by the core counseling skills of Motivational Interviewing (MI) [40]. This counseling approach aims to enhance behavior change through analysis and resolution of ambivalence, a normal process for change [40]. Previous studies have supported the effectiveness of MI for health promotion in individuals of different ages, genders, and ethnicities [41]. Previous interventions based on MI have increased the motivation and self-efficacy to eat Vegetables and Fruits [42]. In our study, the RD blogger sets a positive example for the experience of ambivalence by sharing her own experience in comparing her perceived benefits and her perceived costs or disadvantages to particular behavior change, such as to involve children in the preparation of weekly meals. Each blog post ended with an evocative open-ended question from the RD blogger to initiate change talk with the study participants in the comments’ section of the blog. Each week, the RD blogger wrote empathic feedback messages based on these conversations as ways to address barriers to behavior change.

**Table 1:** Overview of performance and change objectives for mothers addressed in the intervention blog and classified by theoretical domains.

Performance objectives	Theoretical domains			
	Knowledge	Beliefs about consequences	Beliefs about capability	Goals
1. Mothers have Vegetables and Fruits and/or Milk and Alternatives with every meal	Mothers define the concept of the Canadian Eat Well Plate [32].	Mothers express the benefits of consuming Vegetables and Fruits and Milk and Alternatives at every meal.	Mothers express confidence to overcome barriers associated with consuming Vegetables and Fruits and Milk and Alternatives at every meal.	Mothers set the goal of consuming Vegetables and Fruits and Milk and Alternatives at every meal.
2. Mothers plan adequately Vegetables and Fruits and Milk and Alternatives purchase and meal preparation	Mothers list tips to allow efficient planning of everyday meals.	Mothers express positive attitudes towards meal planning and meal preparation.	Mothers express confidence to overcome barriers associated with meal planning and meal preparation.	Mothers set the goal of using a written grocery list to buy all necessary food to prepare planned weekly meals.
3. Mothers make healthy food choices at the grocery store	Mothers understand the nutrition information provided on Canadian food labels.	Mothers express the benefits of reading food labels to make healthier food choices.	Mothers express confidence to overcome barriers associated with efficient use of food labels while purchasing food.	Mothers set the goal of selecting healthier foods at the grocery store based on food labels.
4. Mothers know economic options to increase daily intake of Vegetables and Fruits and Milk and Alternatives	Mothers list economic substitutions to fresh Vegetables and Fruits.	Mothers express the benefits of cooking meals at home with unprocessed ingredients such as Vegetables and Fruits and Milk and Alternatives food groups.	Mothers express confidence to overcome barriers associated with food purchases and home cooking.	Mothers set the goal of cooking a weekly meal with seasonal Vegetables and Fruits. Mothers set the goal of cooking restaurant-inspired dishes or takeout favorites at home.
5. Mothers increase daily intake of Vegetables and Fruits and Milk and Alternatives of the family	Mothers list tasks that can be performed by children in the kitchen.	Mothers express positive attitudes towards involving children in the planning and preparation of meals.	Mothers express confidence to overcome barriers associated with involving children in the kitchen.	Mothers set the goal of making lunches for oneself and children based on the Canadian Eat Well Plate. Mothers set the goal of involving children in the planning and preparation of a weekly meal.
6. Mothers make healthy substitutions in recipes	Mothers list a variety of techniques to cook Vegetables and Fruits. Mothers identify ingredient substitutions to make recipes healthier.	Mothers express the benefits of varying the techniques to cook Vegetables and Fruits, and using ingredients substitutions.	Mothers express confidence to overcome barriers associated with ingredients substitutions.	Mothers set the goal of cooking Vegetables and Fruits with a cooking technique that preserves the nutritional values of food (eg, steaming). Mothers set the goal of adjusting a recipe to make it healthier.

### Step 4: Producing Program Components and Materials

The fourth step of IM protocol involved creating intervention themes, scope, and content sequence by consulting with the intended intervention users to determine preference for intervention program design [17]. We were guided by the results of our preliminary focus group study [19] for creating the sequence and the design of the blog as well as writing blog posts and creating featured recipes. An overview of the blog intervention timeline and components is presented in Multimedia Appendix 1.

#### Creating the Sequence of the Intervention

A 6-month intervention was then developed during which we will address one performance objective per month in weekly blog publications written by an RD. A total of 26 weekly posts will be published. Each weekly post will target alternately one behavioral outcome (increase Vegetables and Fruits intake or increase Milk and Alternatives intake). In a typical month, posts published in the first and third weeks will target the theoretical domains knowledge and beliefs about consequences. The posts published in the second and fourth weeks of each month will target the theoretical domain beliefs about capabilities. We will address the theoretical domain goals (intention) in every post using the theory-based methods prompting goal setting and reviewing of behavioral goals. Multimedia Appendix 1 provides more details on the components and sequence of blog posts throughout the intervention.

**Table 2:** Overview of selected theory-based methods clustered by theoretical domains, parameters for use, and examples of practical applications to the blog^a,b,c^.

Theoretical domains	Theory-based methods	Parameters for use	Examples of practical applications to the blog
Knowledge	Information about health consequences	Messages need to be relevant and not too discrepant from the beliefs of the individual; can be stimulated by surprise and repetition; will include arguments.	The use of the intervention blog itself allowed us to transfer relevant nutritional knowledge, eg, about the health benefits of consuming recommended daily servings of Vegetables and Fruits and Milk and Alternatives food groups.
	Feedback on behavior	Feedback needs to be individual, follow the behavior in time, and be specific.	The RD blogger provided positive feedback on participants’ behavior through comments function of the blog.
Beliefs about consequences (attitude)	Emotional consequences	Present messages as individual and undeniable and compare them with absolute and normative standards.	The RD blogger provided knowledge about the advantages of consuming Vegetables and Fruits and Milk and Alternatives food groups every day and shared real-life examples on how changing these behaviors improved the quality of her eating habits.
	Information about social and environmental consequences	May include awareness about serving as a role model for others.	The RD blogger provided knowledge about the advantages of efficient planning meals, such as reducing meal preparation time and food expenses, and improving the diet quality of their child.
Beliefs about capabilities (self-efficacy, perceived behavioral control)	Verbal persuasion to boost self-efficacy	Credible source.	Through an empathic and positive writing style, the RD blogger told study participants that they could all perform the weekly goals and ascertained that all could increase their daily intakes of Vegetables and Fruits and Milk and Alternatives food groups.
Goals (intention)	Goal-setting (behavior)	Commitment to the goal; goals that are difficult but available within the individual’s skill level.	At the end of each post, the RD blogger encouraged study participants to take up a challenge, such as involving children in meal preparation in the upcoming week.
	Review of behavior goal(s)	Raising awareness must be quickly followed by increase in problem-solving ability and self-efficacy.	At the beginning of every blog post, the RD blogger prompted the study participants to comment on their experience of the previous week’s challenge with an open-ended question.
Social influences (social support, social norms)	Modeling or demonstrating the behavior	Attention, remembrance, self-efficacy and skills, reinforcement of model, identification with model, coping model instead of mastery model.	The RD blogger provided real-life realistic examples of how she overcomes barriers to increase her daily servings of Vegetables and Fruits and Milk and Alternatives. Each post contained a step-by-step recipe featuring Vegetables and Fruits and Milk and Alternatives food groups. Recipes were described textually and with step-by-step pictures.
Skills	Graded tasks	The final behavior can be reduced to easier but increasingly difficult sub-behaviors.	As the intervention moved forward, the sequence of the blog provided an overview of more general aspects of healthy eating (eg, the Canadian Eat Well Plate [32]) to more complex skills (eg, reading food labels).

^a^Theory-based methods were selected from the Basic Methods for Behavior Change from the IM taxonomy [33] and the behavior change techniques from the Behavior Change Technique Taxonomy v1 [34], grouped by the theoretical domains of the Theoretical Domains Framework [25], based on the structure proposed by Cane et al [35].

^b^We judged it appropriate to use behavior change techniques associated with the construct of skills, as the acquisition of real skills is complementary to improvement in self-efficacy for behavior change [34,37]. Given the important social aspect of blogs [38,39], modeling, a behavior change technique associated with the construct of social Influences, was also used.

^C^Parameters for use were drawn from the IM taxonomy [33].

#### Designing the Blog

The intervention blog was developed on the self-hosted blogging platform, WordPress [43]. We worked in collaboration with the Web designer at the Office of the Executive Vice Rector, Development, at Laval University to respect the preferences of female social media users according to our preliminary focus group study [19]. Blog features included a home page composed of a colorful header (graphic and text identifier at the top of every page) and a clear identification of the RD blogger (name, professional picture, expertise and professional credentials); an archiving system of blog posts by date and keywords to facilitate navigation; blog posts divided by subtitles and paragraphs to facilitate reading; internal and external links to references to peer-reviewed scientific papers or other reputable online resources (eg, government websites); and a comments section for interactive communication between the RD blogger and the study participants. The blog also included a Recipe page (recipes published on the blog and categorized by types of meals) and a Resource page providing supplementary healthy eating tools (eg, Health Canada Eat Well Plate [32]). Multimedia Appendix 2 provides screenshots of the intervention blog.

#### Writing Blog Posts and Creating Featured Recipes

One author (A-AD, RD blogger) wrote each post with careful attention to providing positive messages on healthy eating that were adapted to the realities of mothers of preschool and school-aged children. Posts were then reviewed by 3 authors (AL, SL, and SD) to produce a final version.

In line with the results of the preliminary focus group study [19], we included a step-by-step recipe featuring Vegetables and Fruits and/or Milk and Alternatives food groups in every post. Two authors (A-AD, AL) and their families pretested all the recipes. Recipes were described textually and with step-by-step pictures.

### Step 5: Planning Program Adoption

The aim of the fifth step of IM protocol was to plan for the adoption of the intervention. Considerations from blog adoption began as early as the needs assessment, as suggested by Bartholomew et al [17]. The specified intervention adoption outcome was that mothers in the intervention group choose to adopt the intervention as indicated by study compliance, participation, and attrition rates. In order to perform adoption outcome, mothers will have to achieve the following performance objectives: (1) complete more than 70% of study questionnaires, (2) attend over 90% of in-person appointments (compliance rate), (3) assess a minimum of 75% of the blog posts (participation rate), and (4) complete the 6-month intervention and follow-up assessment at 12 months, for a study attrition rate below 25%.

Practical applications were planned in order to stimulate mothers to use the blog. Prior to the beginning of the intervention, 2 authors (A-AD and AL) will provide instructions on how to navigate the blog website and how to post comments to all recruited mothers in order to facilitate their use of the blog throughout the study. Mothers who do not log in to the blog for 2 consecutive weeks will be reminded of the blog by email. As low actual reach, decline usage of online tools, and high attrition rates are common difficulties in Web-based interventions [44], we included engagement-promoting methods identified in previous Web-based lifestyle promotion interventions to promote mothers’ adoption of the blog and adherence to the intervention, as described below.

#### Specifying Engagement-Promoting Methods

Brouwer et al [45] found that key exposing-promoting methods that could increase the participants’ exposure to Web-based healthy lifestyle promotion interventions (eg, more average logins and longer visits on the website) were peer and counselor support, email or phone contacts and updates, and regular updates of the website. In addition, data from our preliminary study showed that women preferred RD bloggers to whom they could relate, as opposed to an unrealistic expert [19]. Recent research suggests that users of narrative health blogs identified more with the bloggers and were more likely to feel they were socially interacting with them compared with users of non-narrative blogs providing expert advice [46]. Consequently, we wrote blog posts and responses to participants’ comments in a narrative approach. By sharing personal experiences involving food, the RD blogger provided a model for mothers to aspire to.

#### Specifying Intervention Characteristics Linked to Enhanced Effectiveness

In addition to engagement-promoting methods, we developed the intervention with special attention to characteristics enhancing effectiveness in Web-based behavior change studies. Webb et al [12] showed that intervention characteristics associated with larger effect size on health-related behaviors in Web-based interventions included more extensive use of theory; incorporating multiple behavior change techniques (eg, modeling, goal setting, and provision of feedback on performance); and using additional methods of communicating with participants, such as providing access to an adviser to request advice. Kohl et al [47] found consistent evidence of effectiveness for tailored feedback, use of theory, interactivity, goal setting, and potentially social support for dietary behaviors. Accordingly, we applied multiple theory-based methods in the experimental blog, as described previously in IM Step 3. In addition to the comments function of the blog, where communication and support between the RD blogger and study participants will be promoted, the research coordinator (AL) will be available by phone or email throughout the study to support mothers with any issues related to the use of the blog website.

### Step 6: Planning for Evaluation

The aim of the sixth step of IM protocol was to plan for an effect evaluation of the intervention, which involves determining whether behavior outcomes change as a result of the intervention [17]. For this task, a randomized controlled trial will measure the effects of the developed intervention blog on dietary behaviors of mothers (Clinical Trial Protocol ID: NCT03156803).

The main objective of the study will be to evaluate the effects of the intervention blog on daily intakes of Vegetables and Fruits and daily intakes of Milk and Alternatives of mothers. As secondary objectives, we will (1) explore the effects of the intervention blog on the dietary habits of mothers’ children, including mean usual food group and nutrient intakes, (2) explore the association between mothers’ child-feeding practices and change in children dietary habits, (3) explore association between intervention blog usage and change in mothers’ dietary habits, and (4) evaluate mothers’ acceptability of the intervention blog.

#### Trial Overview

A total of 110 mothers aged 18 years old or over are being recruited in the Quebec City area of Quebec, using the following strategies: advertisement to a list of people that had indicated their interest to participate in our research institute clinical studies and to email lists of Laval University employers and students; flyers in community centers targeting families, schools, and daycares; and advertisements on Facebook. Mothers will be eligible if they have Internet access, have at least one child aged between 2 and 12 years, are primarily responsible for food purchases and preparation in the household, and consume fewer than the recommended daily serving of Vegetables and Fruits and/or of Milk and Alternatives food groups in Canada’s Food Guide (ie, less than seven servings per day of Vegetables and Fruits (560 g), and/or less than two servings per day of Milk and Alternatives). We sought participants with suboptimal dietary eating habits, and mothers were selected as the target population as they are active blog users [48] and have an important influence on the diet quality of their children [49].

**Figure 3:**
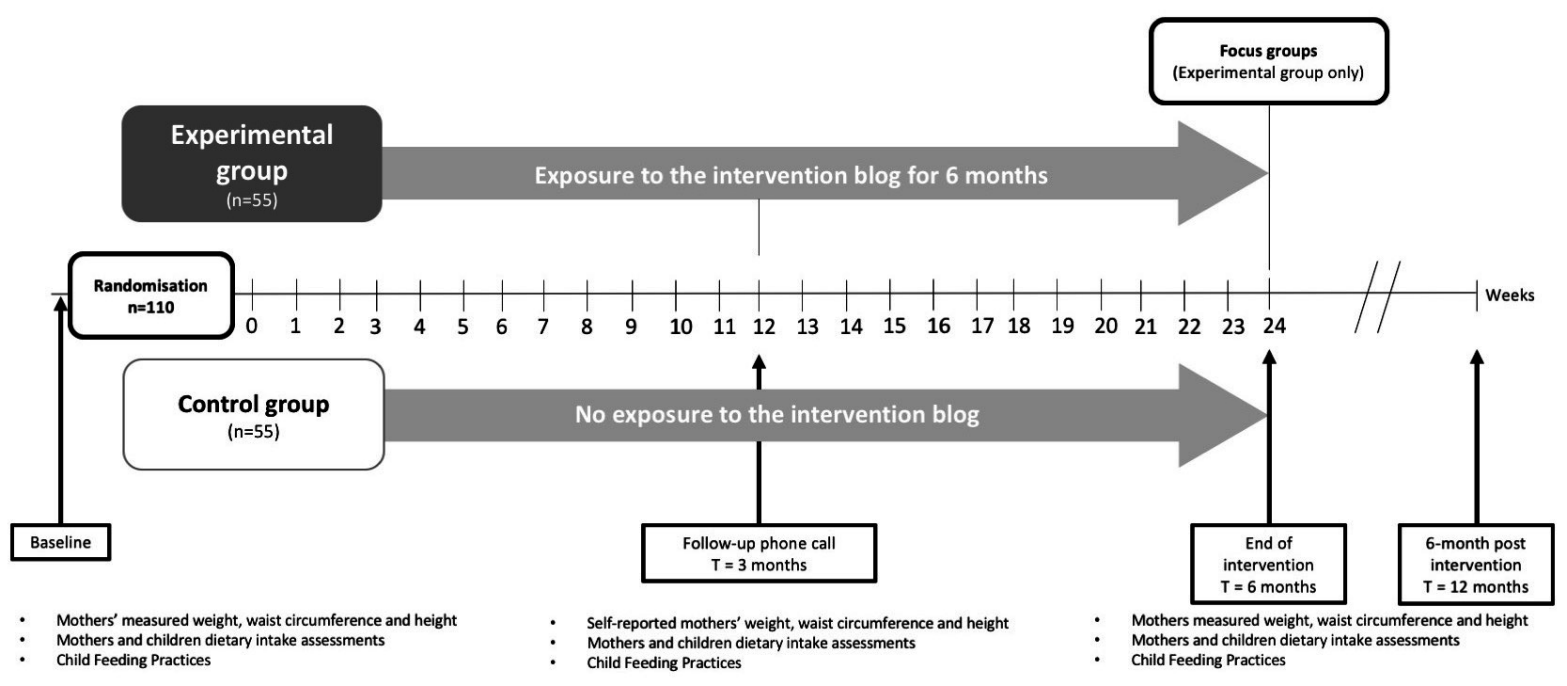
Description of the study and measurements performed at baseline, 3 months, 6 months, and 6 months after the end of the intervention blog exposure.

Eligible mothers will be randomized to the experimental group, which will include access to the blog over a 6-month period, or to a control group with a delayed exposure to the blog (Figure 3). All study participants will complete outcome assessments at baseline, 3 months, 6 months, and 6 months after the end of the intervention (T=12 months). Once baseline testing and the randomization are performed, mothers in the experimental group will receive an email including a unique identification code and a password to log in to the intervention blog and for the research team to collect log data over the intervention. In addition to login information, this email will invite mothers to consult the first published post on the blog through a Web link. Once a week for 26 weeks, mothers will receive by email an alert advertising that a new post has been published. New posts will be dated and displayed in reverse chronological order on the blog home page, making new entries easily traceable. Additionally, posts will be archived by themes, and a user “search terms” feature will be available for mothers to consult previous posts or comments throughout the 6-month intervention. These features have been identified by female social media users in our preliminary focus group study as being useful and likely to increase their use of the blog [19].

The control group will be a delayed control condition. During the 6-month intervention period, mothers in the control group will not be allowed to access the blog. They will not be contacted by the RD blogger but will meet the research coordinator (AL) at our research institute for baseline, 3-month, 6-month, and 12-month outcomes assessment. After the 12-month follow-up, mothers in the control group will be granted access to the blog’s archives containing the RD bloggers’ posts and comments to the posts’ authors by fellow study participants. Interactions with the RD blogger or study participants through the comments function of the blog will not be possible for mothers in the control group.

#### Outcome Measures

Outcome measures for effect evaluation were selected after reviewing the intervention logistic model (Figure 2) with emphasis on the evaluation of mothers’ behavior change.

##### Dietary Variables

The primary outcomes of the study are mothers’ daily servings of Vegetables and Fruits and Milk and Alternatives food groups, which will be assessed by three automated, self-administered, Web-based 24-hour dietary recalls completed by mothers at each assessment time, for two unannounced weekdays and one weekend day selected at random. Multiple administrations of 24-hour dietary recall in prospective studies that aim to determine change between points of time in mean usual intakes of groups are supported by national recommendations [50]. Recent evidence has shown that, for all food groups but the most rarely consumed, two to four dietary recalls were superior to use of Food Frequency Questionnaire data to estimate usual intake [51], and that obtaining 3 nonconsecutive days of 24-hour dietary recalls per month over a period of 6 months was adequate to estimate energy and macronutrient intakes [52]. The Web-based automated 24-hour dietary recall that will be used in this study was developed in French [53] based on the US Department of Agriculture Automated Multi-Pass Method [54] and validated among French-Canadian adults [55]. In addition to data on food groups servings [6], a list of foods and beverages selected by mothers, and macronutrients and micronutrients intakes will be obtained from The Nutrition Data System for Research (software version 4.03, Food and Nutrient Database 31) [56] and the Canadian Nutrition File (version 2015) [57] in order to assess whether changes in mothers’ daily servings of Vegetables and Fruits and Milk and Alternatives food groups will be accompanied by other dietary changes.

Mothers will complete one 24-hour dietary recall administered by an RD with the Automated Multi-Pass Method [54] for their oldest eligible child (aged between 2 and 12 years old) at each assessment time. We have chosen to administer one 24-hour dietary recall at each time point to assess children’s diet as a secondary outcome so as to not overburden mothers with questionnaires and also because the National Cancer Institute’s latest recommendations [50] support the single administration of 24-hour dietary recall to assess change in mean usual intake of a food group between two points in time. The collection of food data by a 24-hour dietary recall has been considered as an appropriate approach with children in previous studies and national nutrition surveys [5,58,59].

##### Mothers’ Child-Feeding Practices

We will assess mothers’ child-feeding perceptions, attitudes, and practices, and mothers’ relationships to children’s developing food acceptance patterns, the control of food intakes, and obesity with the Child Feeding Questionnaire [60] at each assessment time. The Child Feeding Questionnaire is a validated, self-reported 31-item questionnaire that was designed for use with parents of preschool and school-aged children [60]. Maternal child-feeding practices, such as the extent to which mothers control how much, when, and what their child eats, have been shown to impact their child’s development of food-intake controls [61].

##### Blog Usage

In line with our adoption plan of the blog (Step 5), we will explore the associations between the dose of the intervention that mothers in the intervention group received, using blog usage data, and change in their dietary habits. Over the 6-month intervention, we will monitor total number of logins, blog posts viewed, submission of comments, and the most frequently accessed links and blog pages viewed using the Web analytics service Google Analytics [62] and the Web analytics plug-in Slimstat Analytics [63].

##### Acceptability of Blog

We will explore mothers’ acceptability of the blog using focus groups at the end of the 6-month intervention blog exposure. A trained female research coordinator (AL) will use a semistructured interview guided on Patton’s recommendations [64]. A standardized open-ended interview questionnaire will be developed according to three constructs of the Technology Acceptance Model [65]: mothers’ perceptions on utility and ease of use of the blog, and mothers’ attitude (advantages/disadvantages) regarding its use to improve their dietary behaviors.

### Recruitment Procedure

We will perform a three-phase intervention study to reduce the delay between recruitment and the actual start of the intervention. We will conduct each experimental phase subsequently to allow the RD blogger to be entirely dedicated to each virtual social community. In each phase, the nutrition information and recipes in the blog will be identical, with the exception of the interaction in the comments’ section, which will be shaped by the participation in each phase. The three experimental phases will be conducted in different periods of the year (ie, winter through summer; autumn through spring; and spring through autumn), and possible seasonal variations in Vegetables and Fruits food group intakes will be tested for in our statistical model. This study was approved by the Laval University Research Ethics Committee (project n^o^2014-257 A-5 / 12-07-2016)

### Sample Size

Sample size calculations were based on a previous study investigating the effect of a dietary intervention on fiber, vegetables, fruits, and fat intake in free-living adults [66]. It was estimated that a sample size of 82 mothers would allow the detection of a 28% difference in vegetable intakes at 12 months, with a standard deviation of 2.05 in servings of vegetables, a power of 0.95 and a two-sided .05 significance level. Based on attrition rates varying between 10% and 37% in Web-based dietary behavior change interventions [67], we anticipate an attrition rate of 25% and therefore plan to recruit a total of 110 mothers.

## Results

The intervention study is expected to be completed in March 2018.

## Discussion

### Principal Considerations

This study describes the use of the IM protocol to develop an evidence-informed blog to promote healthy eating among French-Canadian mothers of preschool and school-aged children. This study will provide valuable guidance for future researchers, health agencies, and health care professionals who are interested in using this systematic approach to develop a blog as a novel knowledge translation strategy to promote health behavior change.

IM protocol allowed for effective decision making at each step of the planning, implementation, and evaluation of the intervention blog. IM protocol enabled a systematic application of evidence from empirical studies, theories of behavior change, and preliminary research data. Well-designed and effective health promotion interventions should draw on theories of behavior and behavior change to advance an understanding for mechanisms of change [68]. A systematic process is thus a valuable guide to identify determinants of behavioral causes related to the targeted health problem and to select the most appropriate theory-based methods to address the identified determinants to achieve behavior change [17]. In this study, combining performance objectives with selected determinants to create matrices for change was a crucial step of the IM protocol to determine which evidence-based knowledge should be disseminated through the blog.

There is emerging research on adaptation of behavioral interventions for social media delivery [36], and on which behavior change techniques could be applied in digital contexts (eg, goal setting, barrier identification/problem solving, prompt review of behavior goals) [16]; however, these results do not explain how behavior change techniques should be translated into practice applications in online settings (ie, when, where, and in what format they will be effective). Webb et al [12] identified behavior change techniques associated with larger effect sizes in Web-based health interventions. In particular, interventions providing stress management or general communication skills had the largest effects on behavior. Web-based interventions were also more effective when more behavior change techniques were included [12], suggesting that the combination of behavior change techniques may be more effective than using one or two techniques in isolation. The results from a meta-analysis [69] on re-analyzed data from Webb et al [12] supported this hypothesis to some extent. The combination of barrier identification/problem solving with providing rewards for behavior change yielded a synergic effect on behavior change [69]. Future research should focus on practical application methods and evaluate which combinations of theory-based methods would increase effect sizes in social media‒delivered behavior change interventions.

To the best of our knowledge, this is the first publication to describe the development of a social media‒based intervention study using the IM protocol. Previous studies have documented successful use of IM protocol to guide the development of Web-based computer-tailored interventions for health behavior change, such as promoting regular physical activity [70,71] and healthy eating [72]. However, a health intervention delivered through a blog differs from computer-tailored interventions in the use of computer technology and the level of implication from researchers. Computer-tailoring consists of adapting health materials to one specific person through a computerized process based on prior individual assessments [73]. The development of computer-tailored interventions is a time-consuming process, but it requires minimal efforts to carry out once the creation and implementation of the intervention are executed [70]. Blogs, on the other hand, require constant attention to write personalized messages based on users’ comments. Although more time-consuming, this interaction between the blogger and users allow for individualized exchanges and empathic feedback to promote behavior change. As an advantage, blogs may be operated on low-cost Web platforms that are intuitive to navigate and that require minimum programming competencies for the research team.

Blogs are increasingly used by health professionals such as RDs to share information, promote healthy behaviors, and educate and interact with the population and colleagues [74]; however, there is limited empirical evidence on how to design, evaluate, and implement blog-delivered behavioral change interventions. This study therefore has the potential to have a significant reach in the field of social media in dietetic practice by providing the first evidence on the systematic development of an evidence-informed blog written by an RD to promote adherence to dietary recommendations. Blogs facilitate lengthier content-rich conversations whose development can be traced back to the blog [75], unlike other social media tools such as Facebook, which focuses more on relationships, or Twitter, which shares short messages mostly as real-time status updates on timely issues. These conversations can offer social support to blog users [39,76] and include evidence-informed health information, expert opinions, and patient experiences [77]. Additionally, female social media users believe that such blogs make the interaction with an RD more accessible, allow the gain of credible nutrition knowledge and nutritionally balanced recipes, and allow reading of other readers’ comments and links to them [19].

### Conclusions

In conclusion, this is the first study to rigorously describe the use of the IM protocol for the development of an evidence-informed blog to promote healthy eating. IM protocol allowed for effective decision making to produce a novel knowledge translation tool that could be used by health care professionals, such as RDs, to increase adherence to dietary recommendations. A randomized controlled trial is planned to evaluate the effect of the blog on dietary habits and behaviors among French-Canadian mothers of preschool and school-aged children.

## Appendix

Multimedia Appendix 1 Overview of the blog intervention timeline and components.

Multimedia Appendix 2 Screenshots of the intervention blog.

## Abbreviations

IM: intervention mapping
MI: Motivational Interviewing
RD: registered dietitian
