# Supplementary material for: Development of an Evidence-Informed Blog to Promote Healthy Eating Among Mothers: Use of the Intervention Mapping Protocol
Source: JMIR Res Protoc. 2017 May 19;6(5):e92. doi: 10.2196/resprot.7147 (PMC5457529; doi:10.2196/resprot.7147)
Supplement: Multimedia Appendix 1 [file resprot_v6i5e92_app1.pdf]

Multimedia Appendix 1. Overview of the blog intervention timeline and components

Months	Weekly blog posts	Performance objectives	Health behaviors	Theoretical domains	Topics addressed in the blog post
1	Post 1	Mothers have Vegetables and Fruits and/or Milk and Alternatives with every meal	Increase Vegetables and Fruits intake	Knowledge and Beliefs about consequences (attitude)	The Canadian Eat Well Plate
	Post 2			Beliefs about capabilities (self-efficacy, perceived behavioral control)	List of Vegetables and Fruits essentials to store in the kitchen
	Post 3		Increase Milk and Alternatives intake	Knowledge and Beliefs about consequences (attitude)	Myths and realities regarding the Milk and Alternatives food group
	Post 4			Beliefs about capabilities (self-efficacy, perceived behavioral control)	List of Milk and Alternatives essentials to store in the kitchen
2	Post 5	Mothers plan adequately Vegetables and Fruits and Milk and Alternatives purchase and meal preparation	Increase Vegetables and Fruits intake	Knowledge and Beliefs about consequences (attitude)	Meals planning basics
	Post 6			Beliefs about capabilities (self-efficacy, perceived behavioral control)	Practical strategies for efficient meal planning
	Post 7		Increase Milk and Alternatives intake	Knowledge and Beliefs about consequences (attitude)	The global problem of food waste
	Post 8			Beliefs about capabilities	Practical strategies to reduce home food waste

				(self-efficacy, perceived behavioral control)	
3	Post 9	Mothers make healthy food choices at the grocery store	Increase Vegetables and Fruits intake	Knowledge and Beliefs about consequences (attitude)	New findings in the Vegetables and Fruits department of our supermarket
	Post 10			Beliefs about capabilities (self-efficacy, perceived behavioral control)	How to incorporate new Vegetables and Fruits in family meals
	Post 11		Increase Milk and Alternatives intake	Knowledge and Beliefs about consequences (attitude)	Understanding food labels
	Post 12			Beliefs about capabilities (self-efficacy, perceived behavioral control)	Easy steps for efficient food labels reading
4	Post 13	Mothers know economic options to increase daily intake of Vegetables and Fruits and Milk and Alternatives	Increase Vegetables and Fruits intake	Knowledge and Beliefs about consequences (attitude)	Consuming seasonal Vegetables and Fruits
	Post 14			Beliefs about capabilities (self-efficacy, perceived behavioral control)	Healthier and economic grocery shopping
	Post 15		Increase Milk and Alternatives intake	Knowledge and Beliefs about consequences (attitude)	Home-cooked meals
	Post 16			Beliefs about capabilities (self-efficacy, perceived behavioral control)	Practical tips to cook at home like a professional chef!

5	Post 17	Mothers increase daily intake of Vegetables and Fruits and Milk and Alternatives of the family	Increase Vegetables and Fruits intake	Knowledge and Beliefs about consequences (attitude)	Get the Whole Family Cooking
	Post 18			Beliefs about capabilities (self-efficacy, perceived behavioral control)	Simple tasks for children in the kitchen
	Post 19		Increase Milk and Alternatives intake	Knowledge and Beliefs about consequences (attitude)	Healthy balanced lunch planning
	Post 20			Beliefs about capabilities (self-efficacy, perceived behavioral control)	Easy steps for efficient lunch planning
6	Post 21	Mothers make healthy substitutions in recipes	Increase Vegetables and Fruits intake	Knowledge and Beliefs about consequences (attitude)	Overview of various cooking techniques to cook Vegetables and Fruits
	Post 22			Beliefs about capabilities (self-efficacy, perceived behavioral control)	Ingredient substitutions to make recipes healthier
	Post 23		Increase Milk and Alternatives intake	Knowledge and Beliefs about capabilities (self-efficacy, perceived behavioral control)	Save cooking and food allergies
	Post 24			Knowledge, Beliefs about consequences (attitude), Beliefs about capabilities (self-efficacy, perceived behavioral control)	Picky eating in children

	Post 25	Summary	Increase Vegetables and Fruits and Milk and Alternatives intake	Beliefs about consequences (attitude), Beliefs about Capabilities (self-efficacy, perceived behavioral control)	Long-term maintenance of healthy eating behaviors
	Post 26			Beliefs about consequences (attitude), Beliefs about capabilities (self-efficacy, perceived behavioral control)	Summary of the 6 performance objectives addressed throughout the intervention
