# Supplementary material for: Development of an Evidence-Informed Blog to Promote Healthy Eating Among Mothers: Use of the Intervention Mapping Protocol
Source: JMIR Res Protoc. 2017 May 19;6(5):e92. doi: 10.2196/resprot.7147 (PMC5457529; doi:10.2196/resprot.7147)
Supplement: Multimedia Appendix 2 [file resprot_v6i5e92_app2.pdf]

Multimedia Appendix 2. Screenshots of the intervention blog.

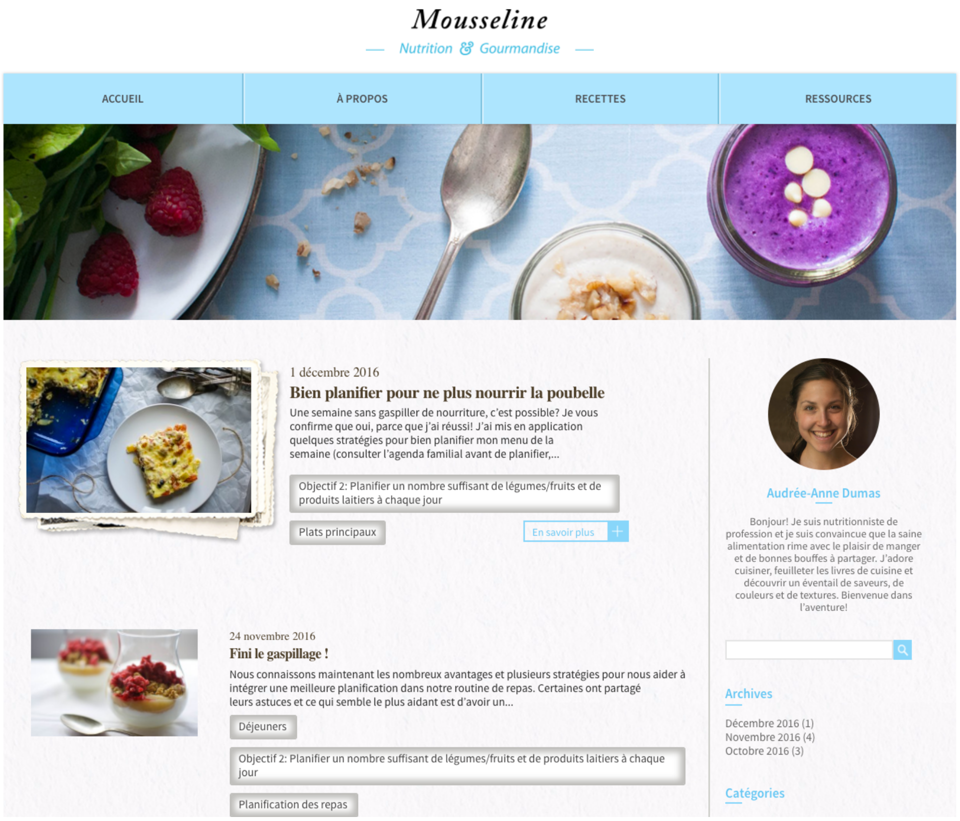

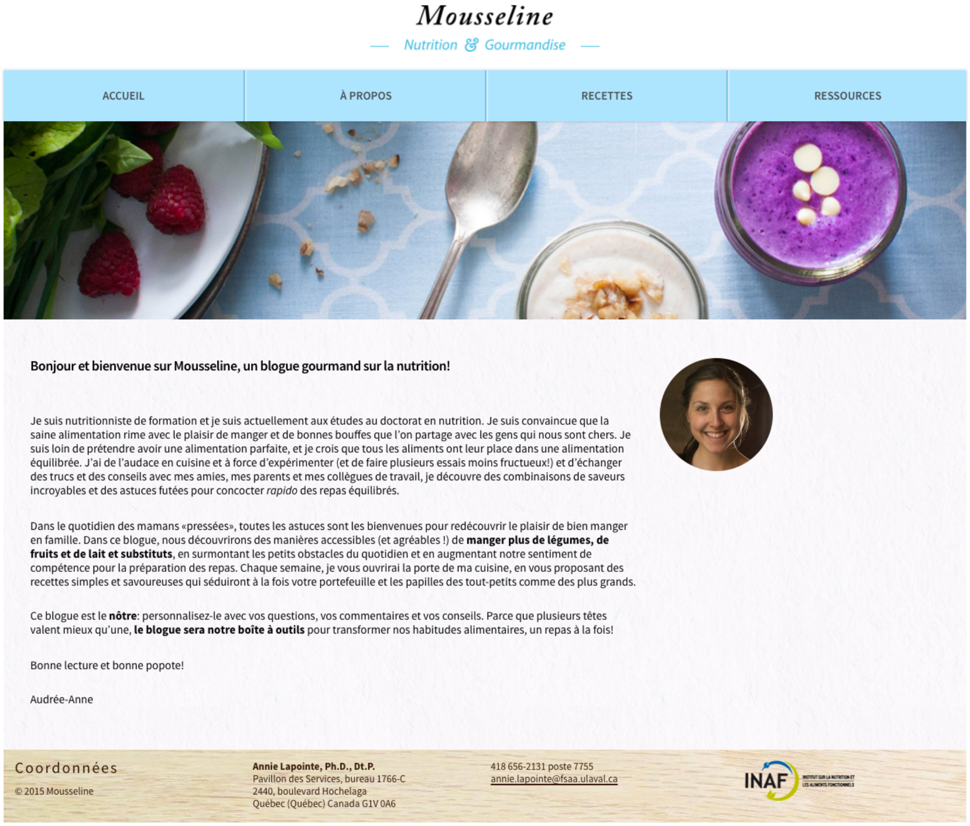

Mousseline

Nutrition & Gourmandise

ACCUEIL | À PROPOS | RECETTES | RESSOURCES

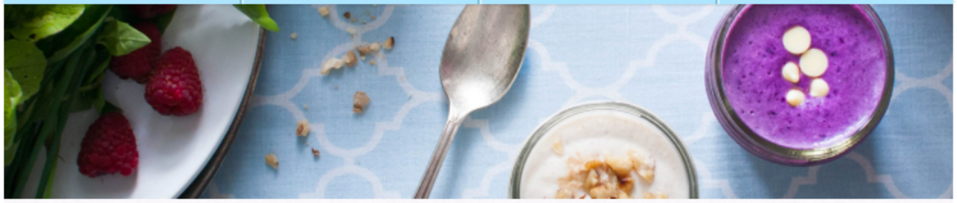

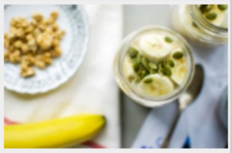

Déjeuners

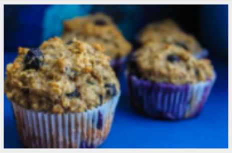

Collations

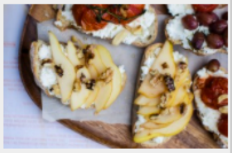

Entrées

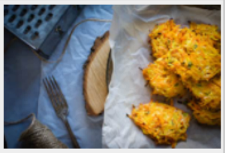

Accompagnements

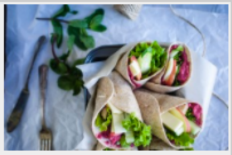

Plats principaux

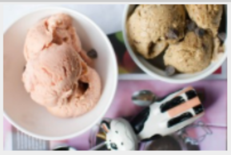

Desserts

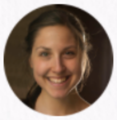

Audrée-Anne Dumas

Bonjour! Je suis nutritionniste de profession et je suis convaincue que la saine alimentation rime avec le plaisir de manger et de bonnes bouffes à partager. J'adore cuisiner, feuilleter les livres de cuisine et découvrir un éventail de saveurs, de couleurs et de textures. Bienvenue dans l'aventure!

Archives

Décembre 2016 (1)
Novembre 2016 (4)
Octobre 2016 (3)

Catégories

Assiette équilibrée
Objectif 1: Consommer des légumes/fruits et des produits laitiers à chaque repas
Objectif 2: Planifier un nombre suffisant de légumes/fruits et de produits laitiers à chaque jour
Planification des repas

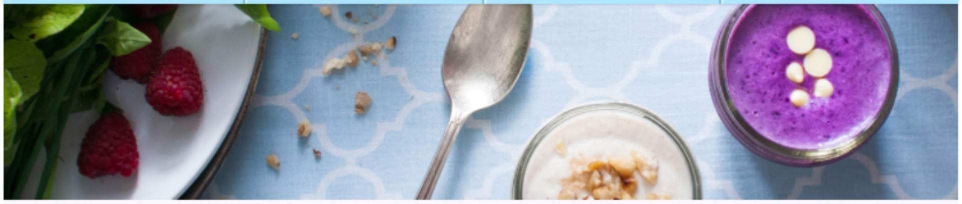

Bien planifier pour ne plus nourrir la poubelle

1 décembre 2016

Objectif 2: Planifier un nombre suffisant de légumes/fruits et de produits laitiers à chaque jour

Plats principaux

Une semaine sans gaspiller de nourriture, c'est possible? Je vous confirme que oui, parce que j'ai réussi! J'ai mis en application quelques stratégies pour bien planifier mon menu de la semaine (consulter l'agenda familial avant de planifier, choisir les repas en fonction du contenu de mon garde-manger, faire une liste d'épicerie, etc.) et j'ai usé de créativité pour réutiliser les surplus de la veille. Ma planification n'était pas parfaite et j'ai dévié un peu de ma liste d'épicerie en me laissant emporter à cause du rabais sur certains légumes. J'ai acheté trop de courgettes et de poivrons la semaine dernière et ils commençaient à flétrir dans mon bac à légumes, alors j'ai préparé une frittata pour ne pas les gaspiller (voir recette ci-dessous!).

> ***Et vous? Comment s'est passé votre défi de faire une liste d'épicerie pour gaspiller le moins possible tout en ayant suffisamment de légumes et de fruits pour en manger à tous les repas cette semaine?*** J'aimerais beaucoup lire vos témoignages.

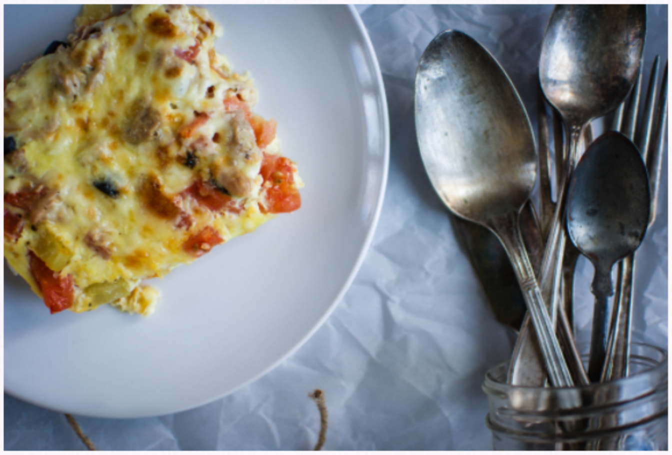

Malgré toutes nos bonnes intentions, il est possible que nous gaspillions de la nourriture à l'occasion. Pour ma part, je jette généralement les aliments parce que mon entourage n'aime pas une nouvelle recette ou parce que je les oublie dans le frigo ou dans le congélateur. Je ne suis pas parfaite et même si ce n'est pas toujours facile, je m'en sors bien grâce à **mes stratégies pour gaspiller moins de nourriture** :

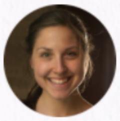

Audrée-Anne Dumas

Bonjour! Je suis nutritionniste de profession et je suis convaincue que la saine alimentation rime avec le plaisir de manger et de bonnes bouffes à partager. J'adore cuisiner, feuilleter les livres de cuisine et découvrir un éventail de saveurs, de couleurs et de textures. Bienvenue dans l'aventure!

Recette

Roulés colorés

Simple et rapide à préparer, cette recette de roulés ajoutera de la couleur et un brin de nouveauté à notre menu de la semaine ! Une belle recette pour utiliser les lentilles en conserve de notre garde-manger et le fromage qui se trouve dans le frigo! Les enfants peuvent aussi participer pour garnir leur roulé.

10 minutes
4 personnes

Ingrédients

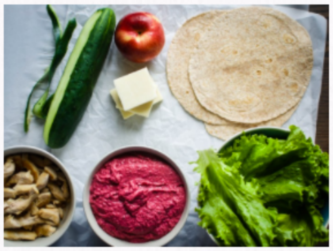

- 250 ml (1 tasse) de houmous de betterave du commerce ou maison (voir astuces)
- 8 petites tortillas de blé entier ou au pesto du commerce
- 8 grandes feuilles de laitue (romaine ou frisée), lavées et asséchées
- ½ concombre moyen avec ou sans la pelure, lavé et taillé en petits bâtonnets
- ½ pomme moyenne avec la pelure, lavée et finement tranchée
- 200 g de fromage cheddar, tranché
- 375 ml (1 ½ tasse) de poulet cuit, tranché en lanières (optionnel)

Préparation

1. Tartiner les tortillas de la préparation de houmous. Garnir d'une feuille de laitue, de bâtonnets de concombre, de tranches de pommes, de fromage et de lanières de poulet si désiré.
2. Rouler les tortillas et servir !

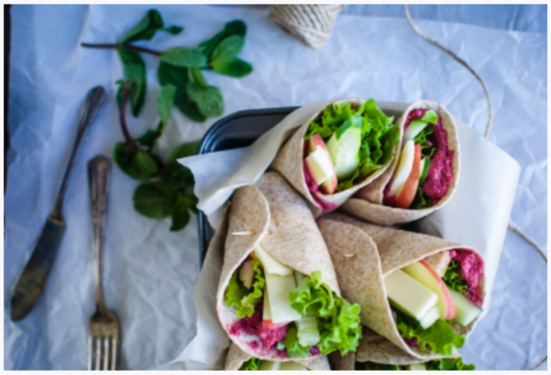

Astuces

- En pique-nique ou dans la boîte à lunch, transportez la tartinade, la garniture et les tortillas séparément et assemblez les roulés à la dernière minute pour éviter de tremper les tortillas.
- Amusez-vous à varier les légumes et les fruits avec de l'oignon rouge tranché, des poires tranchées, des pousses de pois mange-tout ou de la luzerne, des carottes en juliennes, etc. Le houmous peut aussi être préparé sans la betterave si vous n'en avez pas sous la main ou une version à la betterave est également disponible dans les supermarchés.

12 commentaires

xblog064 dit :
17 novembre 2016 à 10 h 58 (Modifier)

Répondre

Je me sers également d'un cartable pour mettre mes recettes préférées que je prends sur internet (Ricardo, recettes Québec, cuisine futée…) . Je conserve mes feuilles de planification de repas, car les mêmes semaines finissent par revenir en rotation. Donc la planification effectuée n'est pas perdue et avec le temps, c'est de + en + facile. Un autre truc avec les enfants est de les impliquer dans la planification. Pour varier le menu, on peut prévoir une nouvelle recette/semaine ou par 2 semaines et demander aux enfants de s'impliquer dans le choix des nouvelles recettes pour éviter l'effet de surprise :«je ne veux pas y goûter». Je fais aussi une batch de muffins, galettes, biscuits ou pain choco/bananes 1 soirée par semaine et je me fais aider des enfants souvent le mardi soir. Les muffins fabriqués me servent ensuite de dessert maison dans les lunchs pour remplacer les barres tendres du magasin. Aussi, mon plus vieux est âgé de 13 ans, alors j'en profite pour qu'il nous prépare un souper de son choix le soir où je fais des commissions ou je règle des r-v, réunions ou encore je m'offre un repos de souper. Il fait une chose simple comme omelette fromage, épinards, saumon fumé ou des pâtes, des bagels… C'est lui qui décide et ça fait toujours partie de ces beaux moments de la journée.

Audrée-Anne Dumas dit :
17 novembre 2016 à 11 h 29 (Modifier)

Répondre

La lecture de votre commentaire me laisse sans mot. Vous nous donnez tellement d'astuces pour faciliter la planification des repas familiaux et comment impliquer les enfants dans le processus ! Votre témoignage inspirera certainement plusieurs d'entre nous pour peaufiner nos compétences de planificatrices. Merci !

Je suis curieuse d'en lire plus sur votre suggestion de confier la préparation d'un souper par semaine à votre garçon de 13 ans. *Comment avez-vous commencé à l'impliquer en cuisine avant de lui confier la préparation d'un repas complet ? Et, est-ce que vous restez présente dans la cuisine pour l'assister ?* Vos réponses nous encadreront pour tenter l'expérience à notre tour !

Mousseline: nutrition et gourmandise 8 0 + Créer Salutations, Audrée-Anne Dumas

Tableau de bord
Accueil
Mises à jour 8
Articles
Médias
Pages
Commentaires
Salient
Apparence
Extensions 7
Utilisateurs
Outils
Réglages
Subscribe2
WP-Activity
SlimStat
Réduire le menu

Options de l'écran ▾ Aide ▾

WordPress 4.6.1 est disponible ! Pensez à faire la mise à jour.

Tableau de bord

D'un coup d'œil

8 articles 5 pages
43 commentaires

WordPress 4.4.5 avec le thème Brooklyn (enfant). Mettre à jour vers la version 4.6.1
Moteurs de recherche refusés

Activité

Publié récemment

Aujourd'hui, 9 h 14	Bien planifier pour ne plus nourrir la poubelle
24 Nov, 9 h 14	Fini le gaspillage !
17 Nov, 9 h 19	Un, deux, trois... planifions !
10 Nov, 9 h 07	Maman, qu'est-ce qu'on mange pour souper ?
3 Nov, 9 h 33	Un p'tit coup de pouce pour en manger plus

Commentaires

Par xblog030, sur Bien planifier pour ne plus nourrir la poubelle
Effectivement, ne pas gaspiller n'est pas toujours facile! Nous avons essayé le planificateur que vous avez proposé. Cela...

Brouillon rapide

Titre

Qu'avez-vous en tête ?

Enregistrer brouillon

Brouillons Afficher tout

Vers la variété et plus loin encore ! 10 mars 2016
[vc_row][vc_column width="1/1"][vc_column_text]L'été est une merveilleuse saison de choix et d'abondance....

À la découverte de nouvelles saveurs ! 17 mars 2016
[vc_row][vc_column width="1/1"][vc_column_text]Est-ce qu'il vous arrive d'être à court d'inspiration en cuisine ?...

L'étiquetage sous la loupe 24 mars 2016
[vc_row][vc_column width="1/1"][vc_column_text]Ce n'est pas le choix qui manque à l'épicerie !...

Mousseline: nutrition et gourmandise 8 0 + Créer Salutations, Audrée-Anne Dumas

Tableau de bord
Articles
Médias
Pages
Commentaires
Salient
Apparence
Extensions 7
Utilisateurs
Outils
Réglages
Subscribe2
WP-Activity
SlimStat

Options de l'écran ▾ Aide ▾

WordPress 4.6.1 est disponible ! Pensez à faire la mise à jour.

Access Log

Filter | est égal à | | Appliquer | Load | 12-01-16 - 12-01-16 ▾

Visitors Activity

Résultats 1 - 44 de 44 [Rafraichir dans 0:52]

aadum4 (10.248.226.115) 1280x800

Un, deux, trois... planifions !	post	SL: 717 / PS: 261	12-01-16 01:57
/	home	SL: 752 / PS: 398	12-01-16 01:57
Maman, qu'est-ce qu'on mange pour souper ?	post	SL: 580 / PS: 261	12-01-16 01:56
Plats principaux	category	SL: 680 / PS: 113	12-01-16 01:56
Recettes	page	SL: 559 / PS: 471	12-01-16 01:55
Bien planifier pour ne plus nourrir la poubelle	post	SL: 597 / PS: 316	12-01-16 01:55
/	home	SL: 742 / PS: 219	12-01-16 01:54
Recettes	page	SL: 535 / PS: 392	12-01-16 01:53
